# Survival and prognostic determinants of prostate cancer patients in Tikur Anbessa Specialized Hospital, Addis Ababa, Ethiopia: A retrospective cohort study

**DOI:** 10.1371/journal.pone.0229854

**Published:** 2020-03-05

**Authors:** Jemal Beksisa, Tewodros Getinet, Sisay Tanie, Jilcha Diribi, Hamid Yimam Hassen

**Affiliations:** 1 Ethiopian Field Epidemiology Training Program, Saint Paul’s Hospital Millennium Medical College, Addis Ababa, Ethiopia; 2 Department of Public Health, Saint Paul’s Hospital Millennium Medical College, Addis Ababa, Ethiopia; 3 Department of Oncology, Tikur Anbessa Hospital, Addis Ababa, Ethiopia; 4 Department of Public Health, Mizan Tepi University, Mizan Teferi, Ethiopia; Northwestern University Feinberg School of Medicine, UNITED STATES

## Abstract

**Background:**

Globally, the incidence of prostate cancer is increasing, particularly in low- and middle-income countries. It is the most common cancer among men worldwide, with higher mortality in low and middle-income countries. In Ethiopia, it is the second most common cause of cancer morbidity and mortality among men. Despite a few studies done regarding the disease burden, the evidence is scarce about the survival and prognostic determinants of prostate cancer patients in Ethiopia. Thus, this study assessed the survival and prognostic determinants of patients with prostate cancer.

**Methods:**

We retrospectively followed patients who were newly diagnosed from 2012 to 2016 at the Oncology Department of Tikur Anbessa Specialized Hospital. We extracted the data from patient charts that were available in the cancer registry using a checklist with the help of oncology nurses. Kaplan-Meier survival analyses with the log-rank test were used to estimate and compare the probability of survival among covariate categories. After checking for assumptions, a multivariable Cox regression analysis was performed to identify prognostic determinants of survival.

**Results:**

The median survival time was 28 months with an overall 2-, 3- and 5-year survival of 57%, 38.9% and 22%, respectively. The overall survival differs according to the clinical stage (P-value<0.01), presence or absence of distant metastasis (P<0.01) and androgen deprivation therapy (ADT) (P<0.05). Cancer stage at diagnosis (adjusted hazard ratio (AHR) = 0.309, 95%CI = 0.151–0.633) and ADT (AHR = 3.884, 95%CI = 1.677–8.997) remained significant in the final Cox proportional hazards model.

**Conclusions:**

The overall 2-, 3- and 5-year survival of prostate cancer patients in Ethiopia is very low. The cancer stage at diagnosis and treatment modalities are significant prognostic determinants of survival. Therefore, early detection through screening and timely initiation of treatment are essential to improve the survival of prostate cancer patients.

## Introduction

Cancer is among the leading causes of premature death worldwide, and the prevalence is still increasing. In 2015, the incidence was estimated to be 17.5 million and was responsible for 8.7 million deaths worldwide [1]. Among all cancer types, prostate cancer is the most prevalent and fifth leading cause of cancer deaths among men in the world, with over 1.44 million patients, 381,000 deaths, and 6.1 million estimated disability-adjusted life years (DALYs) in 2016 [1]. Previously cancer was described as a disease of the riches, but now it has become evident that it is also a public health problem in low- and middle-income countries (LMICs). This could be due to the lifestyle changes, rapid urbanization, cultural transition, and an increase in life expectancy in LMICs. From 2005 to 2015, the highest (10–20%) increase in the incidence of cancer observed in the World Health Organization (WHO) African region [1, 2]. Furthermore, there are prevailing challenges like poor screening and diagnostic practices, minimal treatment options, inadequate research and training, and limited population-based cancer registration, makes the burden of prostate cancer in low-income countries to be underestimated [3–6]. The mortality due to prostate cancer is disproportionately affecting low- and middle-income countries with 165,000 deaths compared to 142,000 in high-income countries [1].

In Ethiopia, similar to other low-income countries, non-communicable diseases including cancer are emerging. Prostate cancer is the third most incident cancer next to breast and cervical cancer and the eighth cause of cancer death in both sexes in 2013 [2]. In 2015, there were 2269 estimated new patients of prostate cancer in Ethiopia [7]. The 2017 Global Burden of Disease (GBD) estimation for Ethiopia indicates prostate cancer caused 1851 deaths and 33,056 disability-adjusted life years (DALYs) [8, 9].

Unlike high-income countries, where patients seek screening and treatment early that lead to a higher probability of survival, patients in low-income countries including Ethiopia present themselves to healthcare late and are expected to have a very limited life span. Goals of care and treatment include; further decreasing the risk of cancer recurrence, alleviating the residual physical and psychological adverse effects of therapy, and improving survival. Estimating the survival rate is necessary to assess the effectiveness and quality of care given to the patients. Albeit a few studies have done describing the disease prevalence, shreds of evidence are scarce on the survival rate and prognostic determinants among prostate cancer patients in Ethiopia. Investigating the survival has important practical value for patients and physicians to understand how the prognosis is changing over time and to decide on better treatment options. Moreover, it enables public health professionals to understand the quality and effectiveness of care and treatments introduced in improving survival and quality of life. Thus, this study primarily aimed to assess the overall 2-, 3- and 5-year survival and to identify its prognostic determinants among prostate cancer patients diagnosed in 2012–2016 at Tikur Anbessa Specialized Hospital, Ethiopia.

## Methods and materials

### Study setting and period

The study was conducted at Tikur Anbessa Specialized Hospital (TASH), a tertiary level teaching hospital with a cancer treatment facility and is the only cancer referral center in Ethiopia. TASH has established the Addis Ababa Population-Based Cancer Registry (AAPBCR) at the radiotherapy center in 2011. AAPBCR is the first population-based cancer registry in the country, which registers cancer patients that are dwellers of Addis Ababa city. The main sources for the population-based cancer registry are hospitals, higher diagnostic clinics, and diagnostic laboratory with pathology services. The starting point for the retrospective follow-up was the time of first confirmed diagnosis of prostate cancer and the endpoint was the date of death, loss to follow up, last contact or the end of follow-up period (December 31/2016).

### Study design and participants

A retrospective cohort study was employed to assess the survival of prostate cancer patients in Tikur Anbessa specialized hospital who were newly diagnosed or referred from January 1, 2012 to December 31, 2016. Patients who first diagnosed prior to January 2012 were excluded from the study regardless of their registration date. Incomplete records or charts having neither of histopathology nor Prostate-Specific Antigen (PSA) level and cancer stage reports were also excluded. We retrieved the charts of all prostate cancer patients using the medical record number obtained from the AAPBCR. Out of 171 prostate cancer patients registered during the study period, 149 charts were retrieved, of which 12 were incomplete (no report of histopathology, PSA level, and cancer stage). Finally, the records of 137 patient charts were extracted and used for analysis.

### Data collection procedures

We used data abstraction form, which was prepared based on the availability of information on patient charts and reviewing the literature on some important variables. Before data collection started, the charts of all prostate cancer patients were identified and retrieved by the medical registration number of the central card room and radiotherapy center of the hospital. Then, data collectors reviewed baseline and follow up characteristics of the patient from the charts. Data that were available in patient records were entered into a data abstraction form manually. To ascertain for the main outcome, as there were no mortality data in the patient charts, a phone call was done to all patients and/or their attendants. During the phone interview, we collected information that was not available from the medical record, including current event status, date of death if died, presence of co-morbidities such as hypertension or diabetes, and other lifestyle factors. We defined an event as the death of patients due to prostate cancer. Patients who have incomplete information on the date of death, were lost to follow up before developing the event, who were died due to other causes unrelated to prostate cancer, and those who have no phone number and whose current status is unknown, were censored to the date of last hospital follow up. While those who did not died until the end of follow-up were censored to December 31, 2016. Oncologic nurses who were working at the cancer treatment center collected the data and facilitated the phone interview. To improve the data quality, a one-day training was given to the data collectors about the objective, methodology, and tools of the study.

### Data processing and analysis

The collected data were checked for completeness, coded and entered into Epi Info version 7.1 and analyzed using STATA version 14.0. There were 21 (15.3%), 45 (32.8%), and 16 (11.7%) missing values for PSA level, histological grade, and imaging result. We assumed, missing data were completely at random, and we, therefore, managed using a multivariate imputation technique [10]. Missing results were imputed for the variables used for the multivariable Cox regression model but not for the outcome variable, death, as we analyzed only participants for whom the outcome was ascertained.

Descriptive statistics including frequencies, percentages, and rates were carried out. The incidence rate of the occurrence of death was also calculated. Kaplan-Meier survival curves were used to show the association between covariates and the time of death, and to describe the differences in the survival rates. To estimate the median follow up time, we used reverse Kaplan-Meier estimator. The Kaplan-Meier curve along with the Log-rank test was used to test for the presence of a significant difference in overall survival among covariate categories. Bivariate Cox proportional hazards regression model was performed to identify variables for multivariable analysis. Variables with p-value 0.2 and less in the bivariate analysis were entered into the multivariable proportional hazards model. Finally, multivariable Cox regression was undertaken for eight variables after checking for the presence of multicollinearity and proportional hazards assumption. Multicollinearity was detected between the variable bone metastasis and distant metastasis, hence the former was excluded from the final model, as it is also one of the distant metastasis. P-values less than 0.05 in the multivariable Cox proportional hazards model were considered as significantly associated with the time to death due to prostate cancer.

### Participant consent and ethical approval

The protocol of this study was approved by the research and ethical committee of Saint Paulo’s Hospital Millennium Medical College. Permission of the medical director and focal person of the cancer treatment center of Tikur Anbessa specialized hospital was obtained. Verbal consent was also obtained from patients or caretakers before starting the phone call interview. The patients’ clinical, laboratory and pathological records were reviewed anonymously and confidentiality of the data was kept at each step of data collection and processing.

## Results

### Socio-demographic characteristics of patients

The age of patients ranges from 43 to 91 years with a median age of 68 years. More than one-third (36.5%) of patients were in the age group between 61 and 70 years. Nearly two-thirds 81 (59.1%) of patients are residents of Addis Ababa city, followed by Oromia region 33 (24.1%).

### Clinical and pathological characteristics of patients

Out of 116 (85%) patients with a tumor marker (pre-treatment serum PSA level) test result, 105 (90%) have a PSA level above 4 ng/ml. Histopathology and imaging test results were available for 92 (67.2%) and 121 (88%) of the patients respectively. Of the 92 patients with a histopathology test report, 89 (96.7%) have adenocarcinoma, 50 (54.3%) of them were poorly or undifferentiated prostate cancer cells. Among a total of 58 (63.0%) of patients with a Gleason score report, 19 (32.8%) have a score of 6 or less, 11 (19.0%) have 7, and 28 (48.3%) have 8 and above. Based on the clinical staging at first diagnosis, 86 (62.8%) and 19 (13.95%) of patients were stage IV and stage III respectively. Out of 81 (59.1%) patients with distant metastasis, 70 (86%), 13 (16%), 6 (7.4%) and 2 (2.4%) involved bone, lymph nodes, liver, and lung respectively. The prevalence of chronic co-morbidities such as hypertension, diabetes mellitus, and HIV infection in our study participants was 13.9%, 10.9%, and 2.9% respectively. One-fifth (19%) of the patients have history of smoking, and only 4 (2.9%) reported a family history of prostate cancer. (Table 1)

**Table 1:** Clinical and pathological characteristics of prostate cancer patients at Tikur Anbessa Specialized Hospital, Addis Ababa, 2012–2016.

Clinical, behavioral and pathological characteristics	Categories	Frequency	Percentage
Age at diagnosis (in years)	< = 50	6	4.4
	51–60	31	22.6
	61–70	50	36.5
	71–80	43	31.4
	> = 81	7	5.1
Serum PSA level (ng/ml) (n = 116)	0–4	11	9.5
	4.1–10	9	7.8
	10.1–20	5	4.3
	above 20.1	91	78.4
Clinical stage at diagnosis	Stage I	10	7.3
	Stage II	13	9.5
	Stage III	19	13.9
	Stage IV	86	62.8
Histological grade (n = 92)	Well differentiated	19	20.7
	Moderately differentiated	23	25.0
	Poorly/undifferentiated	50	54.3
Histological type (n = 92)	Adenocarcinoma	89	96.7
	Sarcoma	2	2.2
	Others	1	1.1
Gleason score (n = 58)	< = 6	19	32.7
	7	11	19.0
	8 and above	28	48.3
Distant metastasis	Yes	81	59.1
	No	56	40.9
Bone metastasis (n = 81)	Yes	70	86.4
	No	11	13.6
Lymph node involvement (n = 81)	Yes	13	16.0
	No	68	84.0
Family history of prostate cancer	Yes	4	2.9
	No	83	60.6
	Unknown	50	36.5
Ever smoking	Yes	26	19.0
	No	111	81.0

## Treatment options given to the patient

Most (81.8%) of patients were treated with Androgen Deprivation Therapy (ADT), while 103 (75.2%) with radiotherapy, 69 (50.4%) received surgery, and 37 (27%) combination of ADT, radiotherapy, and surgery. Of patients who received surgery, radical prostatectomy was done in 58 (84%) of them, and transurethral resection of the prostate (TURP) was performed for 11 (16%). Only 7 (5%) of patients were treated with chemotherapy alone. (Table 2)

**Table 2:** Treatment options given to prostate cancer patients at Tikur Anbessa Specialized Hospital, Addis Ababa, 2012–2016.

Treatment options	Patient received	Frequency	Percent
Androgen Deprivation Therapy (ADT)	Yes	112	81.8
	No	25	18.2
Radiotherapy	Yes	103	75.2
	No	34	24.8
Surgery	Yes	69	50.4
	No	68	49.6
Combination therapy ^a^	Yes	37	27.0
	No	100	73.0
Chemotherapy	Yes	7	5.1
	No	130	94.9

a- Combination of ADT, radiotherapy and surgery.

### Survival from time of diagnosis to death

**Fig 1:**
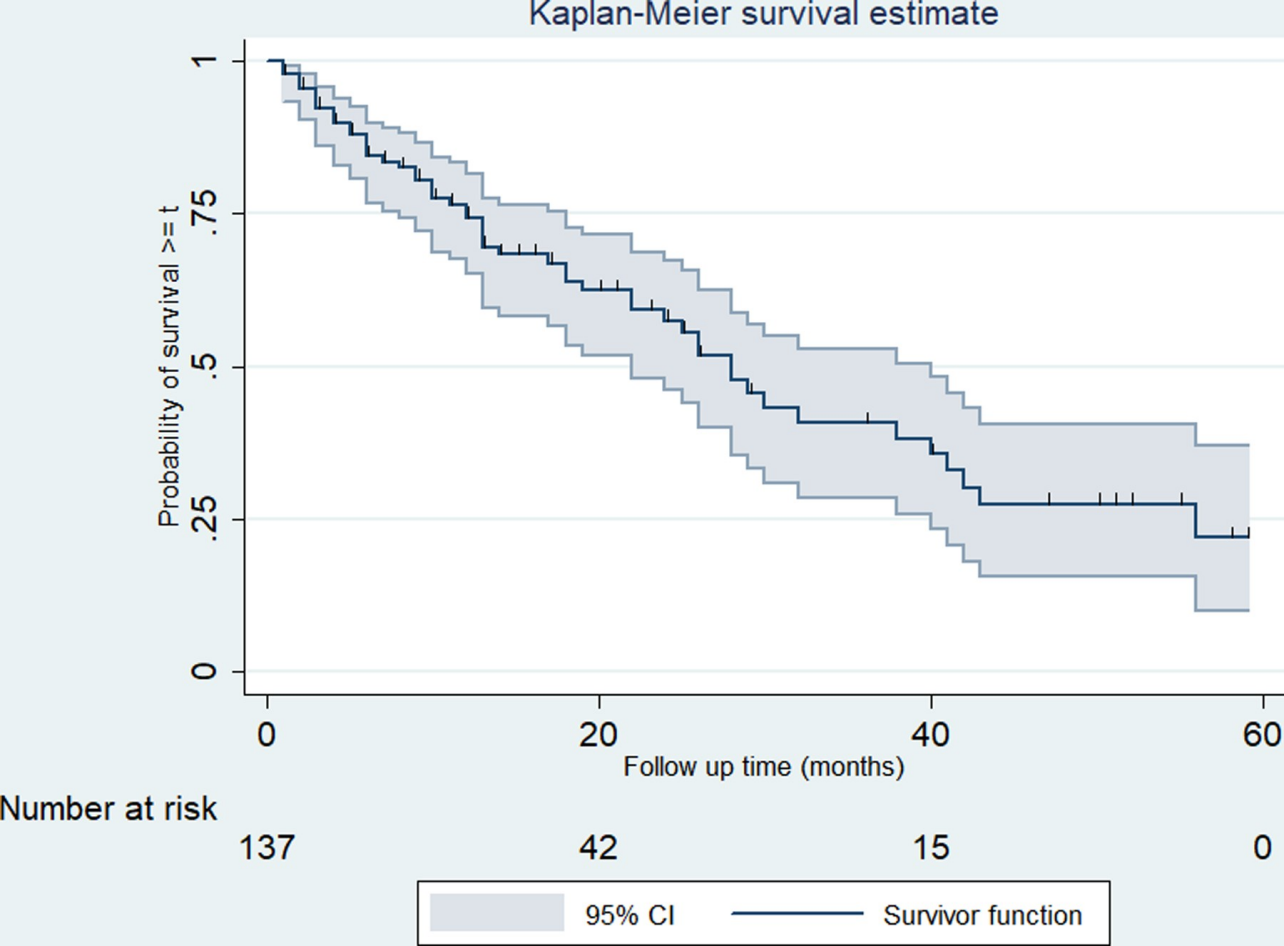
Kaplan-Meier survival curve among prostate cancer patients. The Kaplan-Meier survival curve indicating the overall survival for prostate cancer patients in Tikur Anbessa Specialized Hospital, Addis Ababa, 2012 to 2016. The curve shows the median survival is 28 months.

Fifty-five (40.1%) of patients were found to be dead during the 2200 person-year follow-up period. The median follow up time was 17 months with range of 1 and 59 months. The overall event rate was 25 per 1000 person-months [95%CI: 19.2 to 32.6]. The overall 2-, 3- and 5-year survival rate was 57.1% [97%CI: 43.4 to 76.2], 38.9% [19.3 to 59.6] and 22.1% [0 to 36.7] respectively, with median survival time of 28 months [95%CI: 22 to 40]. (Fig 1)

### Survival rates among different groups of prostate cancer patients

**Fig 2:**
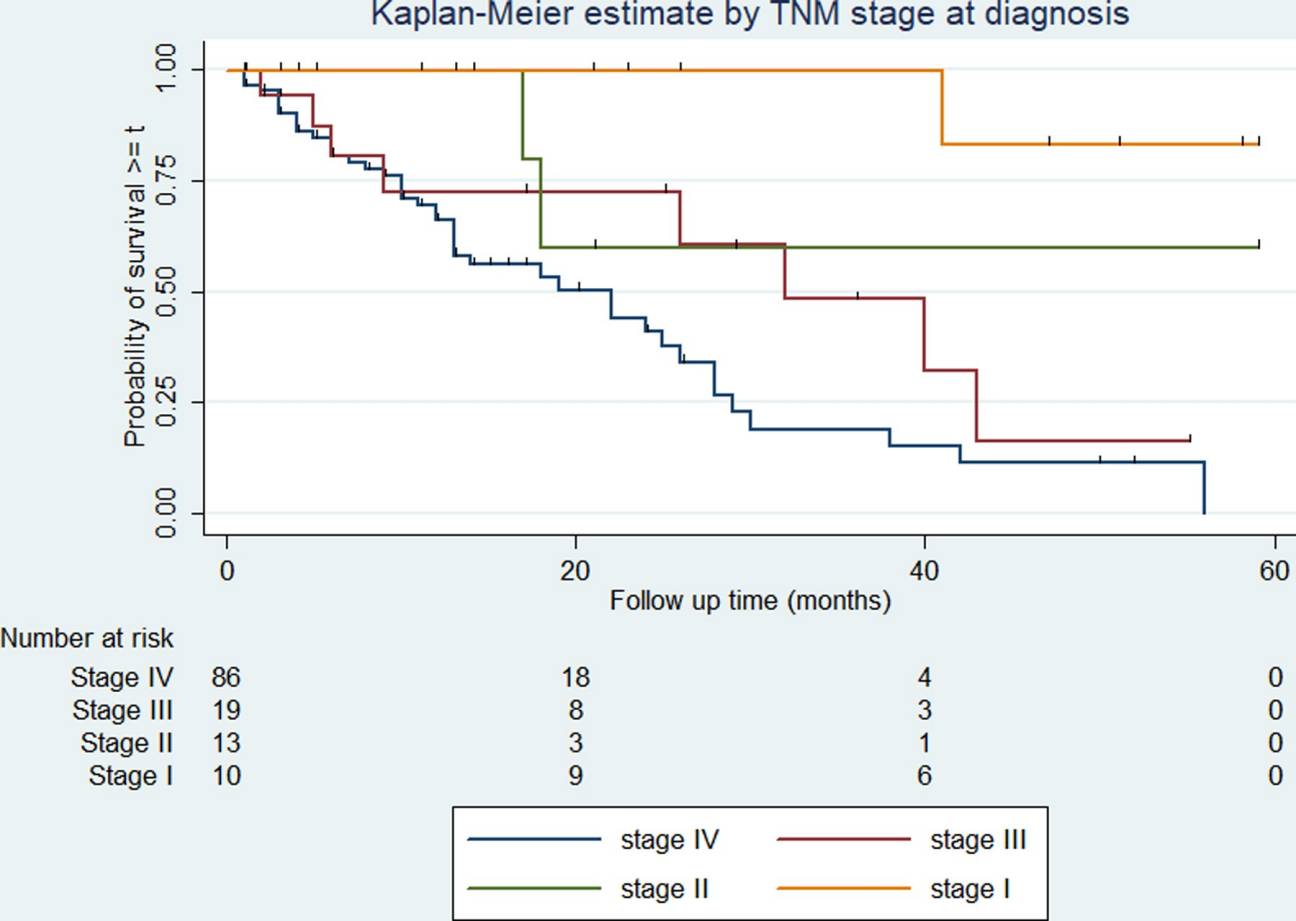
Kaplan-Meier survival curve and stages of cancer among prostate cancer patients. The Kaplan-Meier survival curve indicating the overall survival for each stage of cancer at diagnosis among prostate cancer patients in Tikur Anbessa Specialized Hospital, Addis Ababa, 2012 to 2016. Log-rank test for comparisons of survival curves indicated a significant difference in the mortality for the difference in stage at diagnosis. **p < 0.01.

**Fig 3:**
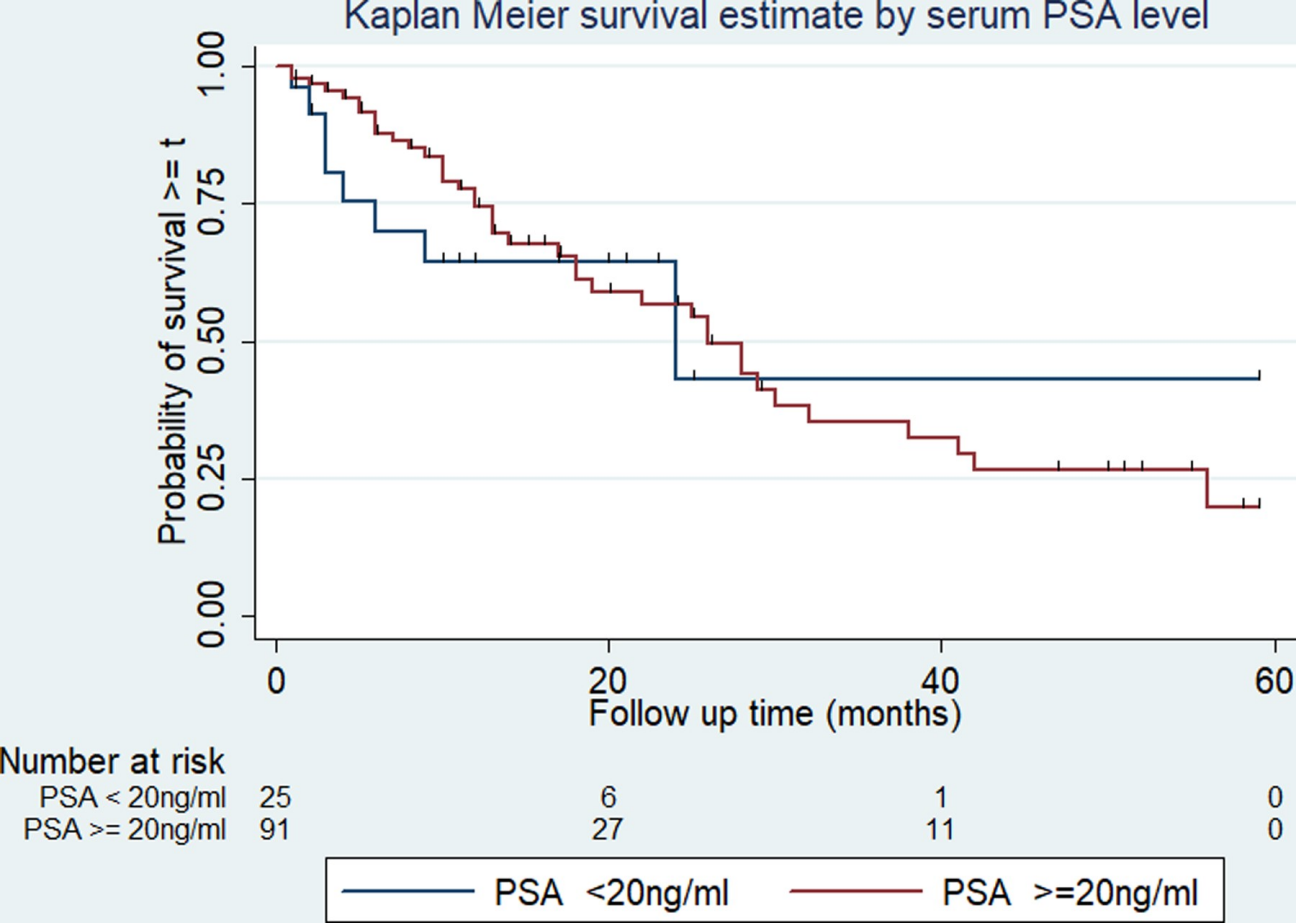
Kaplan-Meier survival curve and serum PSA level among prostate cancer patients. The Kaplan-Meier survival curve indicating the overall survival for serum PSA level less than and above 20ng/ml among prostate cancer patients in Tikur Anbessa Specialized Hospital, Addis Ababa, 2012 to 2016. Log-rank test for comparisons of survival curves indicated a PSA level less than 20ng/ml at diagnosis have better survival than those above. *p < 0.05.

**Fig 4:**
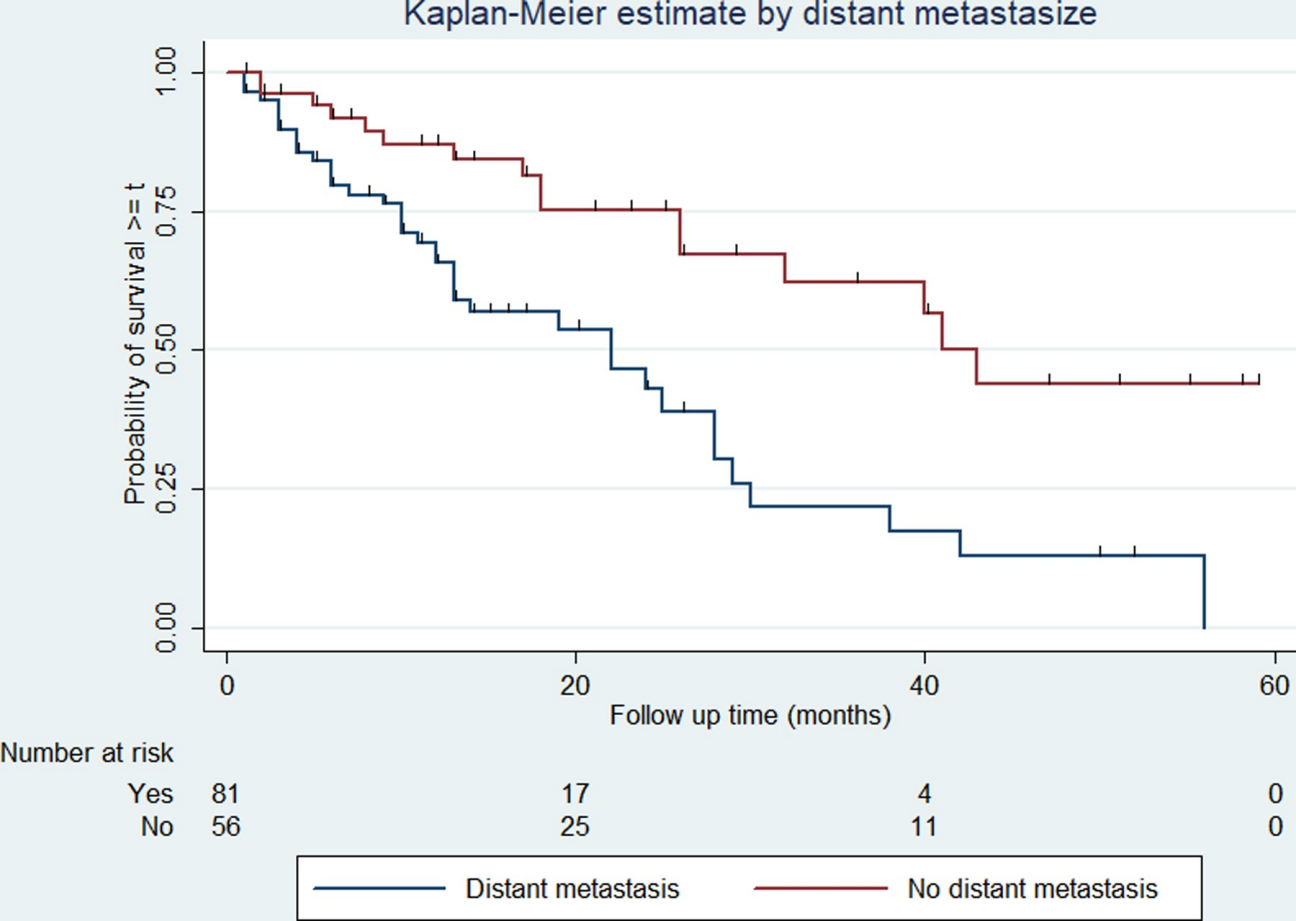
Kaplan-Meier survival curve and the presence of distant metastasis among prostate cancer patients. The Kaplan-Meier survival curve indicating the overall survival for each the presence/absence of distant metastasis at diagnosis among prostate cancer patients in Tikur Anbessa Specialized Hospital, Addis Ababa, 2012 to 2016. Log-rank test for comparisons of survival curves indicated patients who had distant metastasis at the time of diagnosis have lower survival than those without. **p < 0.01.

**Fig 5:**
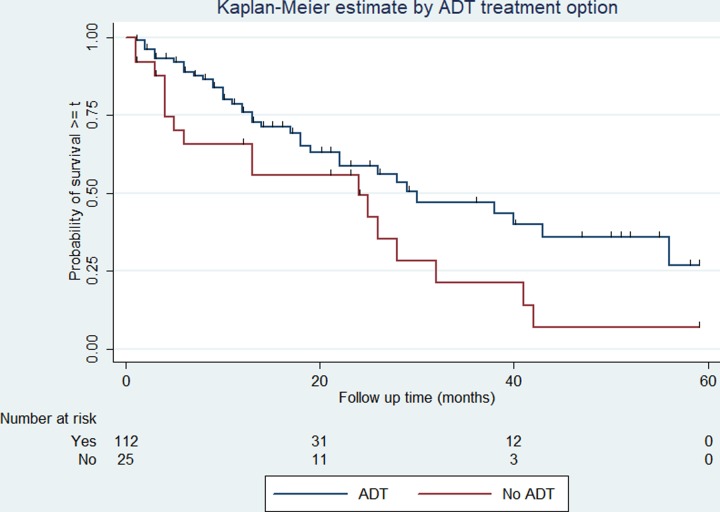
Kaplan-Meier survival curve and ADT treatment among prostate cancer patients. The Kaplan-Meier survival curve indicating the difference in overall survival for treatment with and without hormone among prostate cancer patients in Tikur Anbessa Specialized Hospital, Addis Ababa, 2012 to 2016. Log-rank test for comparisons of survival curves indicated a significant difference in the mortality for the difference in stage at diagnosis. *p < 0.05.

The survival rate varies among categories of covariates such as the cancer stage, presence or absence of distant metastasis, ADT treatment, and serum PSA level. The overall survival rate varies according to the stage of cancer at diagnosis (log-rank test p < 0.01). Similarly, patients who have had a serum PSA level of less than 20 ng/ml at diagnosis have better overall survival compared with those above 20 ng/ml (log-rank test p = 0.048). The 5-year survival is 25% and 19% in patients with PSA levels less than 20mg/ml and 20mg/ml or above respectively. The overall survival varies significantly among patients with and without distant metastasis (log-rank test, p < 0.01). Patients with distant metastasis at the time of diagnosis experienced 2- and 5-year survival of 40% and 0% respectively, while those with no distant metastasis the 2- and 5-year survival was 67% and 44% respectively. Similarly, patients who were treated with ADT (either of orchidectomy or other ADT drugs) experienced 2- and 5-year survival of 58% and 27% respectively, while the corresponding rate for those who were not treated with ADT is 49% and 7% respectively. The variation in overall survival across the determinants is presented in Figs 2–5.

### Prognostic determinants of time to death among prostate cancer patients

Cancer stage at diagnosis and androgen deprivation therapy remained significant in the multivariable Cox proportional hazards model. As the cancer stage is one step higher the probability of death increase by more than 3 fold (AHR = 3.236, 95%CI: 1.580–6.623). On the other hand, the probability of death decreased by 74.3% for patients who received ADT compared with those who do not (AHR = 0.257, 95%CI: 0.111–0.596). (Table 3) However, no interaction effect was observed between any of the predictors.

**Table 3:** Cox proportional hazards model indicating the prognostic determinants of survival among prostate cancer patients at Tikur Anbessa Specialized Hospital, Addis Ababa, 2012–2016.

Variables	Adjusted Hazard Ratio (AHR)	Std. Err.	Z-statistic	95%CI for AHR
Distant metastasis (yes)	1.832	0.934	1.19	(0.674, 4.976)
Age at diagnosis	1.010	0.021	-0.49	(0.970, 1.054)
Histological grade				
Well differentiated	1			
Moderately differentiated	0.780	0.549	-0.35	(0.196, 3.097)
Poorly or non-differentiated	1.368	0.816	0.599	(0.425, 4.403)
TNM stage	3.236	0.113	-3.21	(1.580, 6.623)**
Lymph Node metastasis (yes)	1.144	0.640	-0.18	(0.599, 4.808)
Smoking (yes)	1.037	0.415	-0.09	(0.446, 2.392)
ADT (yes)	0.257	1.665	3.17	(0.111, 0.596)*
Serum PSA level	1.211	0.347	-0.46	(0.532, 2.762)

** significant variable at level of p value 0.01.

* significant variable at level of p value 0.05.

## Discussion

The present study aimed to determine the survival and prognostic determinants of prostate cancer patients who start treatment at Tikur Anbessa Specialized Hospital. We found that the overall 2-, 3- and 5-year survival of prostate cancer patients was very low after they began treatment. This finding is in line with studies done in Nigeria and Sudan, which found 27% and 36.3% of 5-year survival respectively [11, 12]. In our study, the overall survival decreases as the clinical stage at diagnosis is higher, which is coherent with the previous study done in Sudan [12]. Similarly, the study in Ghana indicates a better survival in patients with early stages of the diseases at diagnosis (stage I & II) than advanced stages (III & IV) [13]. However, the overall survival rate in this study is lower compared to the studies done in high-income countries [14, 15]. The observed discrepancies in survival between countries seem to be largely a result of differences in the availability and accessibility of early diagnosis and treatment. The majority of the patients in our study were diagnosed at advanced stages, rather than as precancerous or early cancers. This could be due to the lack of screening programs or confirmatory tests to detect early stages of the disease in low-income countries including Ethiopia. The asymptomatic nature of prostate cancer until it becomes invasive also contributes to a higher number of patients diagnosed at an advanced stage leading to a low survival rate.

Our analysis found a significant difference in survival rates among categories of cancer stages, presence/absence of distant metastasis, bone metastasis, PSA levels at diagnosis, and ADT treatment. Patients diagnosed at early stages have significantly better overall survival than those diagnosed at advanced stages. Survival rates declined steadily with the advanced stage of disease at diagnosis which is also demonstrated by several studies [12, 15]. Moreover, patients with bone metastasis have a lower survival rate compared to those with no bone involvement. This finding is consistent with the study conducted in Indonesia, which compares survival based on a bone scan index measurement and found, patients with high bone scan index values have lower survival [16, 17].

In our study, patients who received ADT have significantly better survival than those who did not, which supports the presumption that ADT is more effective therapeutic option for advanced or metastatic cancer patients than surgery and radiotherapy. On the other hand, patients treated with radiotherapy alone had shown lower survival compared to those who received a combination of ADT, surgery, and radiotherapy. This is supported by a study conducted in Japan, patients treated with a combination of ADT have shown better survival. Furthermore, patients who had a serum PSA level of less than 20ng/ml at diagnosis have better survival compared to those above 20ng/ml. This implies that initiating treatment at an earlier stage leads to a better survival. In clinical practice, a PSA level of 20 ng/ml is used as the cutoff value for advanced prostate cancer and PSA 4 ng/ml is used as a cutoff point for prostate cancer diagnosis [18, 19].

In the multivariable Cox proportional hazards model, earlier stage of cancer at diagnosis and ADT treatment were significantly associated with better survival. Accordingly, the likelihood of death increases by 3 fold as the cancer stage is one step higher. This result is supported by studies in Sweden and the USA that shows patients with higher tumor category have poor survival [20, 21]. This indicates the cancer stage at diagnosis highly determines survival irrespective of age, treatment and other clinical patterns of patients.

In our study, the rate of death due to prostate cancer decreases by 74.3% for patients who were treated with ADT compared with those who did not receive it. The study in Japan and Italy support this result, patients at metastatic stage treated with a combination of ADT showed better overall survival compared with single ADT [22, 23]. This implies that ADT provision, in combination or singly, improves the survival of patients with prostate cancer.

The present study has shown age at diagnosis, presence/absence of distant metastasis, lymph node involvement, histological grade, and smoking status were not statistically significant in the multivariable Cox regression. This contradicts with studies that found these factors as independent significant determinants of survival [24–27]. The reason for this discrepancy could be the biological relationship of these factors along with smaller sample size influenced our findings. Due to smaller sample size, our multicollinearity diagnostic did not identify multicollinearity with these factors.

The use of a robust analytical method is one of the strengths of the present study. A multivariable Cox regression allowed the analysis of survival for patients with an unequal follow-up period and also accounted for censored data. Moreover, we included all the patients who fulfilled the eligibility criteria and this helped us to avoid the risk of sampling error. However, the findings from this study should be interpreted with the perspective of the following limitations. First, due to the nature of medical records, information was not available for some important variables that could affect the multivariable analysis. Second, the ascertainment of death and the cause was based on a phone interview with their family or caretakers. Even though estimation of death affected to a lesser extent, the cause may not be accurate as hospital death records. This may lead to overestimation of deaths due to prostate cancer, which may cause outcome ascertainment bias. However, due to lack of vital event registration system in the country, this is the best available mechanism to ascertain for the cause of death. Since the bias is non-differential, i.e. independent of the determinants under study, the impact on the hazard ratio is minimal. Lastly, due to small sample size, particularly number of events in comparison with the number of variables included to the multivariable model, the estimates could be less precise. Future studies with larger sample size might identify additional determinants of patient survival.

## Conclusion

In conclusion, the overall survival of prostate cancer patients who begin treatment is very low. The survival rate significantly varied among categories of cancer stage, distant metastasis, bone involvement, PSA level, and treatment modalities. The cancer stage at diagnoses and whether the patient received ADT determine the survival of prostate cancer patients independent of other determinants. Lower survival rate was observed for patients who were diagnosed at a late cancer stage and not received ADT, which indicate the key role of early detection and timely initiation of treatment to improve survival and quality of life. Therefore, we recommend improvement and integration of prostate cancer control program, including prevention, screening, and timely initiation of treatment. Furthermore, public health professionals should put effort toward increasing public awareness about prostate cancer screening. Clinicians working at oncology centers should record detailed patient characteristics on the patient chart and the cancer registry form to improve the utility of the data for further research and policy formulation. Further research employing robust research designs with large sample size is recommended. We recommend, a larger prospective study on a cohort of prostate cancer patients that enable researchers to collect as much information as needed.

## Supporting information

S1 Table Sensitivity analysis. Comparison of complete case analysis and multiple imputation for the Cox proportional hazards model indicating the prognostic determinants of survival among prostate cancer patients at Tikur Anbessa Specialized Hospital, Addis Ababa, 2012–2016.
(DOCX)

S1 Dataset This is the minimal dataset of the study. (ZIP)
