# Supplementary material for: Survival and prognostic determinants of prostate cancer patients in Tikur Anbessa Specialized Hospital, Addis Ababa, Ethiopia: A retrospective cohort study
Source: PLoS One. 2020 Mar 5;15(3):e0229854. doi: 10.1371/journal.pone.0229854 (PMC7058322; doi:10.1371/journal.pone.0229854)
Supplement: S1 Table — Comparison of complete case analysis and multiple imputation for the Cox proportional hazards model indicating the prognostic determinants of survival among prostate cancer patients at Tikur Anbessa Specialized Hospital, Addis Ababa, 2012–2016. (DOCX) [file pone.0229854.s001.docx]

|  | **Multiple imputation (MI)** | | | | **Complete case analysis (CCA)** | | | |
| --- | --- | --- | --- | --- | --- | --- | --- | --- |
| **Variables** | **Adjusted Hazard Ratio (AHR)** | **Std. Err.** | **95%CI for AHR** | **P value** | **Adjusted Hazard Ratio (AHR)** | **Std. Err.** | **95%CI for AHR** | **P value** |
| **Distant metastasis (yes)** | 1.832 | 0.934 | (0.674, 4.976) | 0.164 | 2.122 | 1.165 | (0.723, 6.224) | 0.171 |
| **Age at diagnosis** | 1.010 | 0.021 | (0.970, 1.054) | 0.723 | 0.993 | 0.022 | (0.950, 1.038) | 0.760 |
| **Histological grade** |  |  |  |  |  |  |  |  |
| Well differentiated | 1 |  |  |  |  |  |  |  |
| Moderately differentiated | 0.780 | 0.549 | (0.196, 3.097) | 0.715 | 0.757 | 0.535 | (0.189, 3.022) | 0.693 |
| Poorly or non-differentiated | 1.368 | 0.816 | (0.425, 4.403) | 0.471 | 1.329 | 0.801 | (0.408, 4.333) | 0.637 |
| **TNM stage** | 3.236 | 0.113 | **(1.580, 6.623)**** | **0.002** | 3.189 | 0.118 | **(1.530, 6.648)**** | **0.002** |
| **Lymph Node metastasis (yes)** | 1.144 | 0.640 | (0.599, 4.808) | 0.691 | 1.265 | 0.645 | (0.278, 6.713) | 0.702 |
| **Smoking (yes)** | 1.037 | 0.415 | (0.446, 2.392) | 0.803 | 1.031 | 0.525 | (0.380, 2.796) | 0.952 |
| **ADT (yes)** | 0.257 | 1.264 | **(0.111, 0.596)*** | **0.039** | 0.285 | 1.432 | **(0.151, 0.824)*** | **0.046** |
| **Serum PSA level** | 1.211 | 0.347 | (0.532, 2.762) |  | 1.104 | 0.362 | (0.546, 2.835) | 0.324 |

Annex 1: Sensitivity analysis: Comparison of complete case analysis and multiple imputation for the Cox proportional hazards model indicating the prognostic determinants of survival among prostate cancer patients at Tikur Anbessa Specialized Hospital, Addis Ababa, 2012-2016.

*** significant variable at level of p value 0.01*

** significant variable at level of p value 0.05*

*The total number of patients in the MI model is 137 (No. of events=55); while for the CCA is 92 (No. of events 41)*
